# Changes in Temporal and Spatial Patterns of Intrinsic Brain Activity and Functional Connectivity in Upper-Limb Amputees: An fMRI Study

**DOI:** 10.1155/2021/8831379

**Published:** 2021-04-23

**Authors:** Bingbo Bao, Lei Duan, Haifeng Wei, Pengbo Luo, Hongyi Zhu, Tao Gao, Xiaoer Wei, Jing Li, Yuehua Li, Yimin Chai, Changqing Zhang, Xianyou Zheng

**Affiliations:** ^1^Department of Orthopedic Surgery, Shanghai Jiao Tong University Affiliated Sixth People's Hospital, Shanghai 200233, China; ^2^Department of Orthopedic Surgery, Yueyang Hospital, Shanghai University of Traditional Chinese Medicine, Shanghai 200437, China; ^3^Institute of Diagnostic and Interventional Radiology, Shanghai Jiao Tong University Affiliated Sixth People's Hospital, Shanghai 200233, China

## Abstract

**Background:**

Amputation in adults is a serious procedure or traumatic outcome, one that leads to a possible “remapping” of limb representations (somatotopy) in the motor and sensory cortex. The temporal and spatial extent underlying reorganization of somatotopy is unclear. The aim of this study was to better understand how local and global structural plasticity in sensory-motor cortical networks changes temporally and spatially after upper-limb amputation.

**Methods:**

We studied 8 healthy nonamputee control subjects and 16 complete upper-limb amputees. Resting-state MRI (rs-fMRI) was used to measure local and large-scale relative differences (compared to controls) in both the amplitude of low-frequency fluctuations (ALFF) and degree of centrality (DC) at 2 months, 6 months, and 12 months after traumatic amputation.

**Results:**

In amputees, rs-fMRI scans revealed differences in spatial patterns of ALFF and DC among brain regions over time. Significant relative increases in ALFF and DC were detected not only in the sensory and motor cortex but also in related cortical regions believed to be involved in cognition and motor planning. We observed changes in the magnitude of ALFFs in the pre- and postcentral gyrus and primary sensory cortex, as well as in the anterior cingulate, parahippocampal gyrus, and hippocampus, 2 months after the amputation. The regional distribution of increases/decreases in ALFFs and DC documented at 2-month postamputation was very different from those at 6 and 12-month postamputation.

**Conclusion:**

Local and wide-spread changes in ALFFs in the sensorimotor cortex and cognitive-related brain regions after upper-limb amputation may imply dysfunction not only in sensory and motor function but also in areas responsible for sensorimotor integration and motor planning. These results suggest that cortical reorganization after upper extremity deafferentation is temporally and spatially more complicated than previously appreciated, affecting DC in widespread regions.

## 1. Introduction

Not only is complete traumatic limb amputation in adults an emotionally disturbing wound, but it can also develop into a serious medical condition. The traumatic amputation event often causes widespread damage and destruction of skin, tendons, muscles, bones, vasculature, and nerves at the trauma site, and because nerves are axotomized, afferent neurons degenerate, terminal swelling ensues, and regenerative sprouting of the severed axons forms neuromas in the limb stump. This deafferentation also leads to structural and physiological changes in the spinal cord, and some previous studies suggest that a remapping, or reorganization, of limb representations (somatotopy) eventually occurs at the cortical level in motor and sensory networks. These changes are often accompanied by altered perceptual sensations reported by the patient.

Alterations in the sensorimotor cortex of limb amputees have been extensively investigated with various electrophysiological and neuroimaging techniques [1–5]. Almost 90% of amputees report “phantom sensations,” which are described as a vague feeling that the missing upper or lower extremity is still present; some amputees experience severe pain in what seems to be the missing limb [6–9]. Transcranial magnetic stimulation combined with EEG recordings has demonstrated enhanced responses, or neural plasticity, in both motor and somatosensory cortexes in amputees with phantom limb pain [2]. Further, resting-state functional magnetic resonance imaging (rs-fMRI) studies have shown that phantom limb pain is correlated with primary sensorimotor functional remapping after amputation, suggesting that this functional reorganization represents an inappropriate adjustment to deafferentation.

Maladaptive plasticity in sensorimotor networks after nerve deafferentation [10, 11] is one hypothesis to explain the phenomenon of phantom pain. Reorganization of the somatotopic body map in the cortex seems to be an important element in distinguishing painful from nonpainful phantom sensations [10]. However, the widely accepted theory that maladaptive plasticity is the cause of phantom pain has been challenged recently. Rather than plasticity, unusually elevated neural activity in the affected sensorimotor regions of the amputated upper extremity may be responsible for phantom pain [12, 13]. These results may indicate that a more complex and multifactorial cause may underlie phantom pain. Accordingly, studies have also investigated cortical plasticity in amputee patients without phantom pain [5, 14, 15] and found that the alteration in the primary somatosensory cortex is not consistently related to pain symptoms [1].

In the last few decades, neuroimaging techniques like fMRI have been routinely used to detect and analyze functional and anatomical changes in the brain [16, 17], essentially probing the functional architecture of the brain. rs-fMRI is a type of fMRI that provides an estimate of the blood-oxygen-level dependent (BOLD) signal in the brain while subjects are awake, quiet, and not performing any task, and rs-fMRI can provide anatomical specificity of sensorimotor cortex subregional somatotopy [18, 19]. The BOLD signals represent the hemodynamic response to neural activity [20]. The amplitude of low-frequency fluctuations (ALFFs) of the BOLD signal is thought to reflect the magnitude of intrinsic neural activity (local activity of individual regions or voxels) and can provide information about interregional functional connectivity. Thus, rs-fMRI research can reveal neurological processes that occur without external stimulation, and analysis of rs-fMRI signals can reveal information about resting-state connectivity of networks. Only a few studies have analyzed rs-fMRI connectivity and its other metrics such as ALFF to determine whole-brain changes after a traumatic event or in neurological pathology like Parkinson's disease.

In the present study, we used rs-fMRI to investigate local and global brain plasticity in patients with upper-limb amputations. Our aim in this study was to map out how local and global structural and functional connectivity in sensory-motor cortical networks and other brain regions change temporally and spatially after upper-limb amputation. We used two reliable and effective measures of rs-MRI: (1) the ALFF and (2) the degree of centrality (DC), the latter of which reflects the number of instantaneous functional connections between one region and the rest of the brain and thus how much a node influences entire brain areas. These parameters were used to indicate the intensity of spontaneous fluctuations in the BOLD signal at 2, 6, and 12 months after traumatic amputation.

## 2. Methods

### 2.1. Participants

Eight healthy limb-intact man and woman volunteers (control group) were recruited, along with 16 traumatic upper-limb amputees, between the ages of 24 years and 55 years (Table 1). The convenience sample of control subjects was recruited to be balanced by age and sex with the amputee group. A detailed medical history, together with careful examination of head CT scans, confirmed that neither amputees nor controls showed evidence of brain lesions, and their medical history showed no evidence of neurological or psychiatric illness.

All procedures used in this study were approved by the Committee for Medical Ethics of Shanghai Jiao Tong University, Affiliated Sixth People's Hospital, and followed the Ethical Principles for Medical Research Involving Human Subjects (WMA-Declaration of Helsinki). Written informed consent was obtained from all study participants.

### 2.2. Data Acquisition and Scanning Procedures

Scans were acquired on a SIEMENS TRIO 3-Tesla scanner in the Shanghai Sixth People's Hospital, Shanghai Jiao Tong University (SJTU). Anatomical data was acquired using a T1-weighted magnetization prepared rapid acquisition gradient echo sequence with the following parameters: TR = 1900 ms, TE = 2.52 ms, flip angle = 9°, and voxel size = 1m misotropic resolution. Functional data based on the BOLD signal were acquired using a multiple gradient echo planar T2^∗^-weighted pulse sequence, with the following parameters: TR = 3000 ms, TE = 21 ms, flip angle = 90°, imaging matrix = 96 × 96, and FOV = 210 mm axial slices. 40 slices with slice thickness of 2.7 mm and no gap were oriented in the oblique axial plane, covering the whole cortex, with partial coverage of the cerebellum. A total of 240 volumes were collected, during which participants were asked to lie still in a dimmed room with their eyes open. They were explicitly asked not to move any body parts.

### 2.3. rs-fMRI Data Preprocessing

rs-fMRI data of each subject were preprocessed with the software Data Processing Assistant for Resting-State fMRI (DPARSF) [21], which is based on Statistical Parametric Mapping software, SPM8 (Wellcome Trust Centre for Neuroimaging, London, UK; http://www.fil.ion.ucl.ac.uk/spm) and the rs-fMRI Data Analysis Toolkit [22]. Functional image preprocessing was executed according to the following steps: (1) the first 10 volumes were discarded from analysis, since the subjects were adapting to the noise of the scanner and the test situation; this preacquisition period also allowed the scanner to stabilize. The number of time points for this period was not less than 230; (2) slice scan time corrections were made; (3) head movement corrections were made; (4) spatial normalization was done, in which the corrected images were then registered to the T1 structural image, and spatially normalized into the standard Montreal Neurological Institute template (resampled into 3 × 3 × 3 mm^3^); (5) nuisance variables, including white matter signals and head motions, were measured using the Friston-24 model, and cerebrospinal fluid signals were removed by regression; (6) spatial smoothing using a 6 mm FWHM Gaussian kernel was performed; (7) finally, linear trends in the time series were removed.

### 2.4. ALFF Calculations

To study the relationship between functional plasticity after upper extremity deafferentation and resting-state brain functions, we employed ALFF analysis to explore which resting-state networks might be differentially activated in amputees compared to healthy control subjects. ALFF is calculated from the BOLD time course at each voxel [23]. The ALFF calculation was performed identically to that described in the studies of Zang et al. [24, 25]. ALFF was calculated for the traditional low-frequency band (0.01-0.08 Hz).

### 2.5. DC Calculations

DC reflects the strength of connections for a given voxel with all other voxels in the brain and can thus capture its relationship with the whole brain network at the voxel level; it represents a node characteristic of large-scale brain intrinsic connectivity networks [26]. Compared to a binary version of DC, weighted DC provides a more precise centrality characterization of functional brain networks [27]. Therefore, we used weighted DC. Pearson's correlations were calculated between the time courses of each voxel with that of every other voxel in the entire brain. Correlation coefficients with *r* > 0.2 were summed for each voxel, and then, a weighted DC was obtained for each voxel. The threshold of 0.2 was used to eliminate counting voxels that had low temporal correlation. It has been shown that different threshold selections do not qualitatively change the results [28]. Spatial smoothing (FWHM = 6 mm) was carried out after DC calculations, since lack of spatial smoothing may lead to artificial local correlations. We used the following formula for this DC calculation:

(1) $$\begin{array}{c} D=\sum aij,\;j=1,\cdots ,N,i\neq j,aij=\left\{\begin{array}{c} 0,\;aij<0.2,\\ aij,\;aij\geq 0.2. \end{array}\right. \end{array}$$

In line with previous rs-fMRI studies, we removed negative correlations [29]. Since the physiological basis of negative correlations is uncertain, it was not calculated [30–32].

### 2.6. Statistical Analysis

Two-sample *t*-tests were used on the two indices of the BOLD signal (ALFF and DC) separately. The results were corrected for multiple comparisons with a combined threshold of a single voxel (*P* < 0.05) with Gaussian random field (GRF) correction.

## 3. Results

### 3.1. Demographic and Clinical Characteristics of Subjects

Table 1 shows basic demographic and clinical characteristics of the amputees contributing data for this study. The mean age of the 8 healthy control subjects and 16 upper-limb amputees was statistically indistinguishable (37.9 years vs. 41.2 years, *P* = 0.07 > 0.05). Also, the two groups were reasonably well balanced by sex, with 3 females and 5 males in the control group and 5 females and 11 males in the amputee group.

### 3.2. Changes in ALFF Over Time

**Figure 1:**
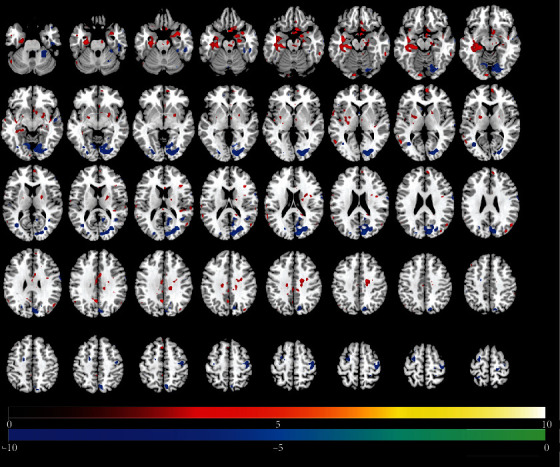
Identification of changed regional brain activity 2 months after traumatic amputation by using rs-fMRI. Relative magnitude of ALFF between upper-limb amputee patients (*n* = 16) and normal control subjects (*n* = 8) is plotted in axial images (top to bottom, inferior to superior). Colors toward red indicate increases in ALFF (significant *t*-values), and colors toward blue indicate significant decreases in ALFF relative to controls (cf. Table 2).

**Figure 2:**
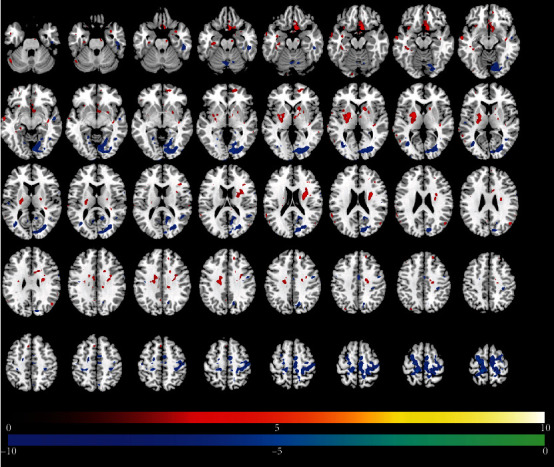
Changes in regional brain activity 6 months after traumatic amputation by using rs-fMRI. Differences in the magnitude of ALFF between the same upper-limb amputee patients and control subjects. Same conventions as in Figure 1.

**Figure 3:**
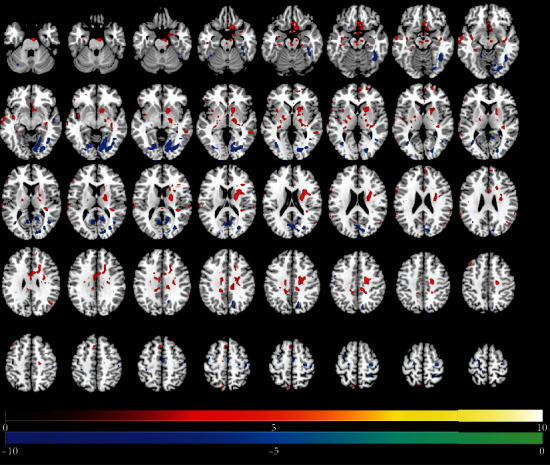
Changes in regional brain activity 12 months after traumatic amputation by using rs-fMRI. Differences in the magnitude of ALFF between the same upper-limb amputee patients and control subjects. Same conventions as in Figure 1.

Tables 2–4 present results of significant regional brain changes in ALFFs at 2-, 6-, and 12-month postamputation, respectively. Figures 1–3 are the corresponding axial images of rs-fMRIs showing relative changes in ALFFs of amputees compared to control subjects.

#### 3.2.1. Two-Month Postamputation

In rs-fMRIs, we observed the largest positive ALFFs in the temporal lobe, cingulate gyrus, pre- and post-central gyri, and precuneus (Table 2, Figure 1). Compared to normal controls at 2-month posttraumatic amputation, in upper-limb amputee patients, we detected significant positive ALFFs in the right side of the brain in the anterior cingulate, parahippocampal gyrus, insula, and putamen, while in the left side at this time point, significant positive ALFFs were detected in the hippocampus, parahippocampal gyrus, putamen, postcentral gyrus, and supplementary motor area. Significant negative ALFFs at 2 months were detected in the right side of the brain in the precentral gyrus, precuneus, and supplementary motor area, while in the left side at this time point, significant negative ALFFs were detected in the precentral gyrus and postcentral gyrus.

#### 3.2.2. Six-Month Postamputation

Six months after the first rs-fMRI scans, a different pattern of regional ALFF changes emerged. In general, the large positive ALFFs we observed in the temporal lobe regions at 2 months were no longer detectable at 6 months or were much reduced in voxel extent (e.g., left hippocampus). One exception was a large relative increase in a negative ALFF in the right supplementary motor area from 42 voxels at 2 months to 903 voxels at 6 months (Table 3, Figure 2). At the 6-month time point, the largest changes were significant negative ALFFs in the paracentral lobule, supplementary motor area, lingual area, and cuneus. Other notable significant positive changes in ALFFs were in the left putamen, right caudate, and negative ALFFs in the fusiform gyrus and inferior temporal lobe.

#### 3.2.3. Twelve-Month Postamputation

In general, at 12-month postamputation, more significant positive and negative changes in ALFFs were detected, but the voxel extents tended to be smaller, and some previously undetected regions emerged at this 12-month scan (Table 4, Figure 3). Exceptions are large positive ALFF changes in the right caudate and left rectus gyrus of the frontal lobe, and large negative ALFF changes in the right lingual area and fusiform gyrus. Interestingly, while the function of the rectus gyrus is unclear, it may be involved in higher cognitive function. We observed the largest positive ALFFs in the cingulate gyrus, pre- and post-central gyri, and precuneus. Persistent significant relative changes in ALFFs were detected in the sensorimotor cortex and some temporal lobe regions (Table 4, Figure 3) across the 2-, 6-, and 12-month scans.

### 3.3. Changes in DC Over Time

**Figure 4:**
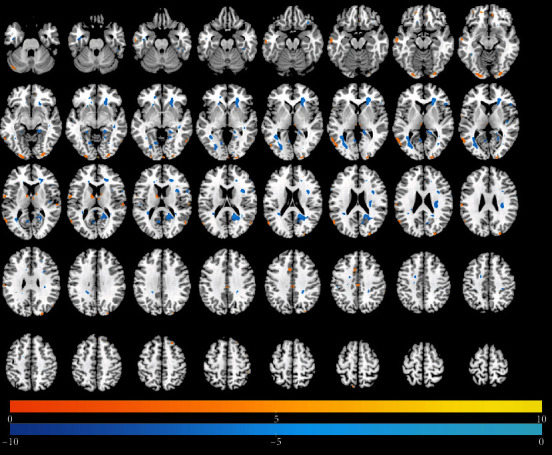
Differences in the magnitude of DC between the same upper-limb amputee patients and control subjects at 2 months of rs-fMRI scan. Same conventions as in Figure 1.

**Figure 5:**
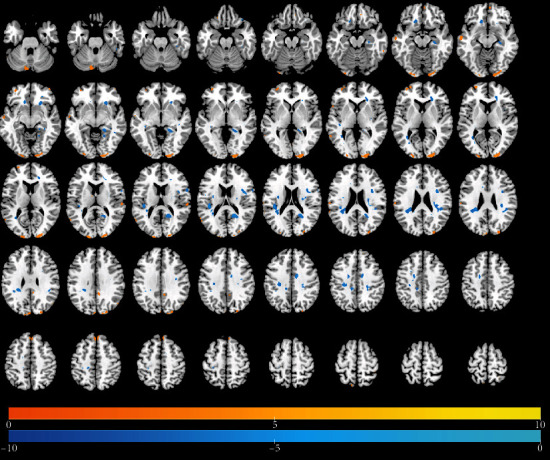
Differences in the magnitude of DC between the same upper-limb amputee patients and control subjects at 6 months of rs-fMRI scan. Same conventions as in Figure 1.

**Figure 6:**
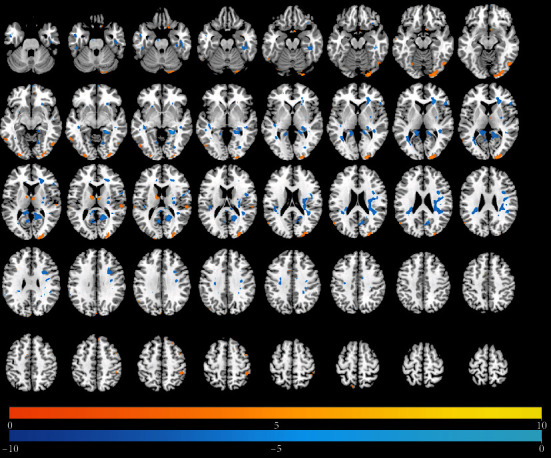
Differences in the magnitude of DC between the same upper-limb amputee patients and control subjects at 12 months of rs-fMRI scan. Same conventions as in Figure 1.

Tables 4–6 present results of significant regional brain changes in DCs at 2-, 6-, and 12-month postamputation, respectively. Figures 4–6 are the corresponding axial images of rs-fMRIs showing relative changes in DCs of amputees compared to control subjects.

#### 3.3.1. Two-Month Postamputation

In rs-fMRIs, we observed the largest positive changes in DC in the left midcingulate gyrus and the largest negative changes in the right precuneus and right parahippocampal gyri (Table 5, Figure 4). In general, the spatial extent of DC changes at 2-month postamputation was much smaller than the corresponding ALFFs at 2 months (cf. Figures 1 and 4). Smaller significant positive or negative changes in DCs were detected in the rolandic operculum, frontal medial orbital cortex, precuneus, parahippocampal gyrus, caudate, midoccipital cortex, and right parts of the triangularis of the inferior frontal gyrus.

#### 3.3.2. Six-Month Postamputation

Six months after the first rs-fMRI scans, a different pattern of regional functional connectivity emerged. At this time point, we detected a new large positive DC in the right calcarine, and other positive and negative DCs were on the same order of magnitude as those observed at 2 months (Table 6, Figure 5).

#### 3.3.3. Twelve-Month Postamputation

At the final scan at 12 months, we detected large positive changes in DC in the right lingual and cuneus and large negative changes in the parahippocampal gyrus and precuneus (Table 7, Figure 6). These changes in functional connectivity either were absent in earlier scans or were much smaller in magnitude.

## 4. Discussion

The temporal and spatial extents of cortical somatotopic reorganization after upper-limb amputation are unclear. In the present study, we described changes in spatial patterns of intrinsic brain activity (ALFFs) and functional connectivity (DC) by measuring rs-fMRI in traumatic upper-limb amputees at 2 months, 6 months, and 12 months. Relative to ALFFs and DC in normal controls, selective increases occurred in the sensory and motor cortex, as expected, but also increases and decreases occurred in related brain regions involved in functional plasticity after upper extremity deafferentation. The regional distribution of increases/decreases in ALFFs and DC documented at 2-month postamputation was very different from those at 6 and 12 months.

The sensory and motor brain networks of the human brain are somatotopically organized. A drastic upper extremity injury in humans, like traumatic amputation, may change the somatotopy of the primary motor cortex and primary sensory cortex of the deafferented hemisphere. Denervation due to amputation or nerve injury disrupts normal sensorimotor function. It is reported that cortical reorganization in the sensorimotor cortex, where cortical maps of intact body parts “expand” into areas associated previously with afferents of the missing limb, also has been reported in numerous animal studies of experimental amputation [33–35].

Sensorimotor reorganization is also observed in some transcranial magnetic stimulation studies in amputees. In these studies, increased excitability in motor areas contralateral to the amputated limb occurs. Remaining stump muscles have higher response amplitudes that can be evoked by transcranial magnetic stimulation applied in a larger area on the scalp than those responses in the intact arm [36, 37]. In addition, a shift in the somatotopy of the lip [38], chin [39], and shoulder [40] representation into the deafferented cortical hand area of upper-limb amputees has been reported using magnetoencephalography (MEG) and fMRI.

Denervation does not lead to a complete loss of the affected limb cortical representation, since the sensorimotor cortex still seems to be working with so-called “attempted movement” by the amputee. When an amputee tries to “move” their phantom limb, activation in the corresponding sensorimotor areas appears in fMRI, and this activation is similar to real executed movements in able-bodied subjects [38, 41, 42].

It has been shown that the persistent cortical representation of upper-limb amputees is relatively detailed for the postcentral gyrus and parietal cortex. For example, in a tetraplegic patient, movement trajectories and movement goals have been successfully decoded from electrophysiological activity in the posterior parietal cortex using intracranial recordings [43]. A persistent hand representation in S1 was also reported in a long-term spinal cord injury patient as measured by microstimulation [44]. Also, the topography of a persistent individual finger representation of a phantom hand has been shown in the somatosensory cortex of amputees [45].

ALFF measures the amplitude of time series fluctuations at each voxel, and DC represents the large-scale intrinsic connectivity of the brain at the voxel level. These measures of rs-fMRI probe brain activity from different perspectives. In our investigation, we found that the ALFF and DC overlap spatially, which mainly occurred in the left superior frontal gyrus (Frontal_Sup_L). This result may suggest that changes in cognition may occur in amputation patient, and may have an extended impact on the brain plasticity.

We also found that the ALFFs in the primary motor cortex and primary sensory cortex decreased at 2, 6, and 12 months after the amputation. The brain regions involved with sensorimotor integration increased, specifically in the putamen, caudate, and precuneus. We speculate that this could represent a compensatory pattern after amputation. The deactivation of sensorimotor cortex may influence the associate cortex of sensory feedback. Since DC values decreased in the precuneus, caudate, and postcentral gyrus, this may indicate that sensorimotor integration is also impaired after limb amputation. It is expected that the primary motor cortex and the primary sensory cortex were silent after the deafferentation of the injured limb. Afterwards, the connection in the sensorimotor network at the affected hemisphere was diminished. In general, we found that amputation patients had significant brain function alterations in local regions and extensive alterations in widespread brain regions, such as corpus striatum and cognition-emotion cortex.

This study had several limitations. First, the sample size of the normal controls and amputation patients was relatively small, which may reduce confidence in the results. Therefore, more subjects would be recruited in future research to definitively evaluate these findings. Second, we chose ALFF and DC as the indices to detect the fluctuations in regional spontaneous activity strength. According to these two measures, the brain of amputation patients showed large-scale changes over 12 months. To clarify the underlying neural mechanisms related to amputation-related brain changes, future studies should examine alterations at the brain network level. Finally, clinical assessment with validated neuropsychological instruments should be performed in future studies, which could uncover a more interesting relationship between amputation and the changed BOLD signals.

## 5. Conclusion

Our findings show that local and extensive temporal and spatial changes occur in intrinsic cortical activity and functional connectivity in the sensorimotor and cognitive-related brain regions after limb amputation. With future confirmatory research, this plasticity may imply that dysfunction occurs not only in sensory and motor regions but also in areas responsible for sensorimotor integration and motor planning. Regardless, these results and others' on how ALFF and DC are plastic after traumatic limb amputation highlight how analyzing intrinsic brain activity in the absence of any task-related sensory or cognitive stimulus (rs-fMRI) may be a fruitful approach for analyzing plasticity after “natural deafferentation” that occurs in Alzheimer's disease or Parkinson's disease.

## Figures and Tables

**Table 1:** Demographic and clinical characteristics of patients with upper-limb amputation (*n* = 16).

No.	Sex	Age (yr)	Amputated side (dominant side)		Amputation level (% limb remaining)		Elapsed time after amputation (mo.) for rs-fMRI scans		
1	M	35	R	(R)	P	(16)	2	6	12
2	M	33	L	(R)	P	(25)	2	6	12
3	M	49	L	(R)	P	(20)	2	6	12
4	F	45	R	(R)	P	(30)	2	6	12
5	F	47	R	(R)	D	(45)	2	6	12
6	F	46	R	(R)	D	(72)	2	6	12
7	M	24	R	(R)	P	(28)	2	6	12
8	M	40	R	(R)	D	(66)	2	6	12
9	M	29	R	(R)	D	(58)	2	6	12
10	F	55	L	(R)	D	(50)	2	6	12
11	F	49	R	(R)	P	(18)	2	6	12
12	M	48	L	(R)	D	(65)	2	6	12
13	M	35	R	(R)	D	(44)	2	6	12
14	M	30	L	(R)	D	(40)	2	6	12
15	M	50	L	(R)	D	(34)	2	6	12
16	M	44	L	(R)	P	(12)	2	6	12

M: male; F: female; L: left; R: right; D: distal; P: proximal.

**Table 2:** Relative regional changes in ALFFs at 2 months^∗^ in upper-limb amputees (*n* = 16) compared to normal control subjects (*n* = 8).

Direction of change	Brain region	Voxels^∗^	*t*-value	MNI coordinates		
				*x*	*y*	*z*
Positive ALFF	Hippocampus_L	189	4.257	-21	-27	-12
	ParaHippocampal_L	189	3.452	-21	-9	-30
	Cingulate_Ant_R	100	3.295	3	27	0
	ParaHippocampal_R	49	4.258	18	0	-21
	Putamen_L	34	3.489	-27	-15	3
	Insula_R	19	3.583	42	-9	-9
	Putamen_R	16	3.334	27	3	-3
	Postcentral_L	11	3.414	-63	-21	18
	Supp_Motor_Area_L	8	3.729	-3	21	54
Negative ALFF	Precentral_R	97	-4.332	42	-12	57
	Precuneus_R	51	-4.492	6	-66	54
	Precentral_L	51	-4.128	-27	0	60
	Postcentral_R	44	-3.836	27	-42	63
	Supp_Motor_Area_R	42	-3.705	3	-15	72
	Postcentral_L	19	-3.758	-21	-45	69

^∗^rs-fMRI scan done 2-month postamputation; MNI: Montreal Neurological Institute; ^∗^number of voxels exceeding threshold (cluster size).

**Table 3:** Relative regional changes in ALFFs at 6 months^∗^.

Direction of change	Brain region	Voxels	*t*-value	MNI coordinates		
				*x*	*y*	*z*
Positive ALFF	Putamen_L	88	4.993	-21	-3	3
	Hippocampus_L	36	3.804	-30	-21	-21
	Caudate_R	17	3.924	15	15	3
	Putamen_R	11	3.386	24	6	-6
	Supp_Motor_Area_L	10	3.521	-3	24	54
Negative ALFF	Paracentral_Lobule_L	903	-6.797	-15	-36	63
	Supp_Motor_Area_R	903	-5.979	6	-6	60
	Lingual_R	428	-5.184	6	-72	-9
	Cuneus_R	428	-4.449	9	-81	24
	Precuneus_R	86	-5.02	15	-66	39
	Fusiform_R	47	-3.948	24	9	-45
	Precentral_R	30	-5.233	48	-3	42
	Temporal_Inf_R	27	-3.21	42	-57	-9

^∗^rs-fMRI scan done 6-month postamputation.

**Table 4:** Relative regional changes in ALFFs at 12 months.

Direction of change	Brain region	Voxels	*t*-value	MNI coordinates		
				*x*	*y*	*z*
Positive ALFF	Caudate_R	202	3.91	15	12	3
	Rectus_L	202	3.835	3	30	-18
	Cingulate_Mid_L	95	4.198	-12	-42	42
	Cingulate_Ant_R	69	3.1	3	18	21
	Frontal_Sup_Medial_R	41	3.936	6	57	27
	ParaHippocampal_L	32	3.362	-27	-39	-12
	Cingulate_Mid_R	30	6.424	12	-33	42
	Thalamus_R	30	3.805	15	-9	-3
	Precuneus_L	28	4.674	-9	-69	60
	Pallidum_L	25	4.531	-21	-3	0
	Occipital_Mid_R	24	3.614	51	-66	24
	Caudate_R	18	5.593	21	-3	18
	Supp_Motor_Area_L	16	4.509	-3	21	54
	Hippocampus_R	14	4.155	36	-30	-6
	Putamen_L	12	3.368	-27	-15	3
	Insula_R	10	3.873	39	-18	0
Negative ALFF	Lingual_R	476	-6.267	18	-63	-6
	Fusiform_R	102	-5.381	24	3	-45
	Lingual_L	67	-3.192	-12	-75	-6
	Precuneus_R	55	-4.646	24	-45	6
	Postcentral_R	41	-6.032	27	-42	63
	Precentral_R	29	-6.454	30	-21	63
	Precentral_L	26	-4.588	-21	-18	63
	Supp_Motor_Area_R	25	-5.477	3	-9	57
	Paracentral_Lobule_L	13	-3.299	-18	-30	66

^∗^rs-fMRI scan done 12-month postamputation.

**Table 5:** Relative regional changes in DC at 2 months.

Direction of change	Brain region	Voxels	*t*-value	MNI coordinates		
				*x*	*y*	*z*
Positive DC	Cingulate_Mid_L	27	3.373	-6	12	39
	Frontal_Med_Orb_R	12	3.29	9	42	-12
	Rolandic_Oper_L	11	4.459	-63	-3	12
	Postcentral_L	10	3.603	-63	-21	27
Negative DC	Precuneus_R	105	-4.736	15	-48	21
	ParaHippocampal_R	105	-3.788	18	-39	-6
	Caudate_R	23	-4.711	21	24	-6
	Precuneus_L	23	-3.808	-9	-60	15
	Occipital_Mid_L	18	-4.159	-27	-78	0
	Frontal_Inf_Tri_R	10	-3.876	51	24	6
	Precentral_R	10	-3.426	60	9	15

**Table 6:** Relative regional changes in DC at 6 months.

Direction of change	Brain region	Voxels	*t*-value	MNI coordinates		
				*x*	*y*	*z*
Positive DC	Calcarine_R	103	5.182	18	-99	0
	Cuneus_L	19	6.229	0	-90	33
	ParaHippocampal_L	14	3.29	-18	3	-33
	Postcentral_L	9	4.074	-63	-21	24
	Postcentral_R	6	3.138	45	-42	63
Negative DC	Precuneus_R	31	-4.937	21	-51	18
	ParaHippocampal_R	27	-4.193	21	-39	-6
	Hippocampus_R	15	-4.685	27	-21	-15
	Precuneus_L	15	-4.31	-3	-54	69
	Cingulate_Mid_R	14	-5.296	12	-6	39
	Cingulate_Mid_L	11	-3.602	-12	-33	42
	Postcentral_L	6	-3.025	-30	-33	54
	Caudate_R	5	-4.04	21	24	-6

**Table 7:** Relative regional changes in DC at 12 months.

Direction of change	Brain region	Voxels	*t*-value	MNI coordinates		
				*x*	*y*	*z*
Positive DC	Lingual_R	188	6.163	24	-93	-15
	Cuneus_R	188	5.813	18	-99	9
	Fusiform_L	23	4.308	-21	3	-45
	Parietal_Inf_R	21	3.916	45	-39	54
	Caudate_L	12	4.725	-12	-3	15
	Postcentral_L	10	4.03	-21	-30	78
Negative DC	ParaHippocampal_R	162	-8.29	21	-39	-6
	Precuneus_R	162	-4.969	12	-63	24
	Cingulate_Post_L	56	-6.593	-6	-39	12
	Fusiform_R	47	-4.827	39	-33	-18
	Precuneus_L	25	-5.595	-6	-60	15
	Frontal_Inf_Oper_R	20	-4.427	36	6	27
	Frontal_Inf_Tri_R	16	-5.115	51	24	6
	Putamen_R	12	-3.446	30	-6	6

## Data Availability

The present study includes 8 healthy volunteers of 5 men and 3 women (mean age: 37.9 years old) and 16 patients with amputation (11 men and 5 women; mean age: 41.2 years old). All procedures used in this study were approved by the Committee for Medical Ethics of Shanghai Jiao Tong University Affiliated Sixth People's Hospital. Written informed consent was obtained from all study participants.
